# The Synergistic Effects of Polysaccharides and Ginsenosides From American Ginseng (*Panax quinquefolius* L.) Ameliorating Cyclophosphamide-Induced Intestinal Immune Disorders and Gut Barrier Dysfunctions Based on Microbiome-Metabolomics Analysis

**DOI:** 10.3389/fimmu.2021.665901

**Published:** 2021-04-22

**Authors:** Rongrong Zhou, Dan He, Jing Xie, Qingyijun Zhou, Hongliang Zeng, Hongmei Li, Luqi Huang

**Affiliations:** ^1^ School of Pharmacy, Changchun University of Chinese Medicine, Changchun, China; ^2^ National Resource Center for Chinese Materia Medica, China Academy of Chinese Medical Sciences, Beijing, China; ^3^ Hunan Academy of Chinese Medicine, Hunan University of Chinese Medicine, Changsha, China; ^4^ Insitute of Chinese Materia, China Academy of Chinese Medical Sciences, Beijing, China

**Keywords:** American ginseng, immunostimulant, gut barrier function, gut microbiota, fecal metabolites

## Abstract

Cyclophosphamide (CTX), used in cancer chemotherapy, a high dose of which would cause immunosuppressive effect and intestinal mucosa damage. American ginseng (*Panax quinquefolius* L.) has a long history of functional food use for immunological disorder, colitis, cancer, and so on. This study aimed to illustrate the underlying mechanism of American ginseng’s immunomodulatory effect in CTX-induced mice. In this study, all groups of American ginseng (American ginseng polysaccharide [AGP], American ginseng ginsenoside [AGG], co-treated with American ginseng polysaccharide and ginsenoside [AGP_AGG]) have relieve the immune disorder by reversing the lymphocyte subsets ratio in spleen and peripheral blood, as well as stimulating CD4^+^T cells and IgA-secreting cells in small intestine. These three treatment groups, especially AGP_AGG co-treated group recovered the intestine morphology that up-regulated villus height (VH)/crypt depth (CD) ratio, areas of mucins expression, quantity of goblet cells, and expression of tight junction proteins (ZO-1, occludin). Importantly, the microbiome-metabolomics analysis was applied in this study to illustrate the possible immuno-modulating mechanism. The synergistic effect of polysaccharides and ginsenosides (AGP_AGG group) restored the gut microbiota composition and increased various beneficial mucosa-associated bacterial taxa Clostridiales, Bifidobacterium, and Lachnospiraceae, while decreased harmful bacteria Escherichia-Shigella and Peptococcaceae. Also, AGP_AGG group altered various fecal metabolites such as uric acid, xanthurenic acid, acylcarnitine, 9,10-DHOME, 13-HDoHE, LysoPE15:0, LysoPC 16:0, LysoPI 18:0, and so on, that associated with immunometabolism or protective effect of gut barrier. These results suggest AG, particularly co-treated of polysaccharide and ginsenoside may be used as immunostimulants targeting microbiome-metabolomics axis to prevent CTX-induced side effects in cancer patients.

## Introduction

*Panax quinquefolius* L. (AG, American ginseng) as one of the *Panax* species, is native to Canada and eastern America and also largely exported to China (1). Both *in vitro* and *in vivo* studies have suggested that American ginseng can be applied as dietary supplement in modulating immune system and used against cancer, viral, and bacterial infection (2). Its active ingredients include polysaccharides (3, 4), ginsenosides (5), and polyacetylenes (6) isolated from AG root. CVT-E002, an aqueous extract from American ginseng (*Panax quinquefolius* L.), which mainly contains polysaccharides, stimulated proliferation of normal mouse spleen cells, and macrophage cytokine production *in vitro*, as well as activated immunoglobulin G production *in vivo* (7). Also, many data have shown that ginsenosides can improve immune function so as to fight against cancer through improving cytotoxicity of natural killer cells and activation of cytotoxic T lymphocytes (8–11). Moreover, it is suggested that AG polysaccharide and ginsenoside can exert their immunoregulatory effects on the gut-associated lymphoid tissue (GALT), while ginsenoside consumption increased the number of jejunal IgA^+^ cells and CVT-E002 increased the IL-1β production in ConA-stimulated mesenteric lymph nodes cells (12). Previous studies indicated AG could restore the dysfunction of gut microbiota in DSS-induced colitis model (13). The combination therapy of AG berry and *Scutellaria baicalensis* could ameliorate side effects of cisplatin-induced vomiting and nausea (14).

Cyclophosphamide (CTX), a widely used chemotherapy medicine, can lead to tumor death due to genotoxicity and cytotoxicity (15). However, patients who take CTX might experience adverse effects, such as vomiting, diarrhea, and abdominal pain, related to disruption of mucosal barrier, bacteria translocation, and change of microbial composition (16). Also, CTX is a potent immunosuppressive agent which may result in occurrence of recurrent infection as well as interfere with wound healing (17). It is suggested to take CTX with other dietary supplements to reduce the side effects. However, it is still unclear whether AG-modified mucosal immunity and gut microbiota contribute to relief of side effects from chemotherapy.

Human gut is colonized by up to 100 trillion of microbes, which maintains a mutual benefit relationship between host and gut microbiota (18). Gut provides nutrients and breeding environment for gut microbiota, and gut microbiota provides benefit in nutrition, metabolism (e.g. vitamin synthesis, carbohydrate fermentation) and immunity. Studies on germ-free mice showed that gut microbiota plays an essential role in mucosal immunity development (19) and host immune response. The presence of specific bacteria species can develop certain sub-types of lymphocytes that shift immune response (20). Mucosal barrier is essential to protect against toxins and invasion of microbes, which can maintain the symbiotic relationship (21). Once the balance is broken, the intestine immune system can lead to diseases, such as inflammatory bowel disease (19). Hence, according to previous superior effects of AG on mucosal immunity and gut microbiota, we hypothesis AG might target gut microbiota to stimulate the immune system.

In this study, CTX-induced immunosuppressive model was established with C57BL/6 mice. The immunoenhancing and the epithelium protection effects of AG were evaluated on immune organ lymphocyte indexes and intestinal morphology analysis, administered 2 weeks of AG ginsenosides and polysaccharides. 16s RNA gene sequencing and UPLC-TOF analysis methods were adopted to detect the variation of gut microbiota and to filtrate the differential metabolites that reveal the underling mechanism. Research results suggest that AG, particularly co-treated of polysaccharide and ginsenoside, have immunoenhancing and the epithelium protection effects in cancer patients. Its mechanism may associate with the gut microbe-metabolic axis.

## Materials and Methods

### American Ginseng Polysaccharide and Ginsenoside Preparation

AG root was purchased from Guangdong Letaotao Pharmaceutical Co., Ltd (Lot. 181203). The 4-year old AG root was collected in Jilin province, China and further identified by associate Prof. Hao Liu (Hunan Academy of Chinese Medicine, China). AG polysaccharides and ginsenosides were obtained from water extracts. In brief, AG powder (100 g) was extracted with 1,000 mL deionized water for 2 h (twice) at 100°C. The extraction solution was concentrated at 60°C and further added with ethanol to 70% ethanol content for overnight. The supernatant was collected as AG ginsenosides solution and dried with vacuum drying technique. AG polysaccharides solution was prepared by alcohol-precipitation and was deproteinized using Sevag reagent (chloroform: n-butyl alcohol = 4:1, v/v). Decoloration was conducted by a D-101 macroporous resin separation method (22). Solution was further deproteinized using Sevag reagent (chloroform: n-butyl alcohol = 4: 1, v/v). Furthermore, AG polysaccharides solution was dried by vacuum drying technique. The qualification and authentication of the AG ginsenosides and polysaccharides were performed using Liquid chromatography-mass spectrometry (LC-MS) and common anthrone-sulfuric acid method, respectively (**Table S1**). The extraction rate of total polysaccharide and ginsenosides were 8.24% (8.24 g polysaccharide extract/100 g American ginseng crude drug) and 2.14% (2.14 g ginsenosides extract/100 g American ginseng crude drug), respectively.

### Animals and Experimental Design

A total of 42 male C57BL/6 mice (5 weeks of age, body weight 20 ± 2 g) were purchased from the Hunan SJA Laboratory Animal Co. Ltd., (Changsha, China, SCXK<Xiang>2019-0004) and housed in the Animal Center (Changsha, China, SYXK<Xiang>2020-0008) under controlled light conditions (12 h light−dark cycle). Mice were provided with food and water ad libitum. After 1 week of acclimatization, mice were randomly divided into six groups with seven mice each: normal control (CTRL) group, CTX-treated group (Baxter International Co., Ltd, Illinois, USA), AG polysaccharides (AGP, 1500 mg/kg/d) group, AG ginsenoside (150 mg/kg/d, AGG) group, AG polysaccharides (1500 mg/kg/d)+ ginsenoside (150 mg/kg/d, AGP_AGG) group, positive control (80 mg/kg/d, LH: Alladdin, Shanghai, China) group. During the experiment, the normal and CTX groups were gavaged with saline, and the other four groups were gavaged with AG polysaccharides, AG ginsenosides, AG polysaccharides, ginsenosides combination, and Levamisole hydrochloride, respectively for 14 days. On the 7th, 9th, and 11th day, all mice except for the normal group were treated with an intraperitoneal injection of CTX (60 mg/kg. bw) once a day for 3 days, while the normal group was treated with same dose of saline. Fresh feces were collected on the 14th day, immediately frozen in liquid nitrogen, and stored at −80°C until further analysis. At the end of the experiment, mice were anesthetized with pentobarbital sodium (60 mg/kg. ip) and sacrificed (23). The blood, spleen, thymus, small intestine, and large intestine were excised for future experiments. All procedures were approved by the Institutional Animal Care and Use Committee (IACUC) of the Institute of Chinese Medicine, Hunan Academy of Chinese Medicine (Hunan, China) and performed in accordance with the Regulations of Experimental Animal Administration (Order No.2, Approved by the State Council in 1988, Third revision in 2017) issued by the State Committee of Science and Technology of the People’s Republic of China.

### Thymus and Spleen Index

The thymus and spleen were dissected and weighed to calculate the thymus and spleen indices. Thymus or spleen index (mg/g) = thymus or spleen mass (mg)/animal body mass (g).

### Hematological Analysis

Blood sample was collected from the eyes and added with EDTA-2K for anti-coagulation. Blood counts were detected by a hematology analyzer (BC-5000Vet, Mindray, Shenzhen, China). The analysis included white blood cells (WBC), lymphocytes (Lym#), neutrophils (Neu#), basophils (Bas#), eosinophils (Eos#), monocyte (Mon#), red blood cell (RBC), hemoglobin (HGB), platelet count (PLT), and mean hemoglobin concentration (MCHC).

### Flow Cytometry Assay

Spleen cells were prepared through a 70-mm nylon mesh strainer and washed with PBS. According to a previous reference of Huyan et al. (24), in short, both spleen suspension and blood sample were stained on ice for 30 min with the following five anti-bodies (Biolegend, San Diego, USA), respectively: PE anti-mouse NK1.1 for natural killer cells analysis, PerCP anti-mouse CD19 for B lymphocyte analysis, APC anti-mouse CD3, FITC anti-mouse CD8a, and Brilliant Violet 421 anti-mouse CD4 for T lymphocyte subset analysis. The percentages of NK cells, B cells, and T-cell subsets were analyzed by a Dxp Athena flow cytometry (Cytek, CA, USA). Ten thousand events were acquired into the list-mode, and staining data were acquired on Flowjo CE software (Tree Star, OR, USA).

### Histological Analysis

The small intestinal tissues were prepared for histological analysis using the methods described by Meng et al. with some modification (25). In brief, after being embedded in paraffin, samples of jejunum tissue were fixed in 4% paraformaldehyde, and then sliced in 4-µm thickness.

In order to measure villus height and crypt depth, tissue were stained with hematoxylin and eosin (HE) after deparaffinization. The images were taken by a light microscope (Nikon Eclipse Ci-L, 40× magnification). The top of the villus to the crypt transition was counted as villus length, while the crypt depth was recorded as the invagination between two villi. The intestinal villus length and crypt depth were measured in each sample *via* Image Pro Plus 6.0 software (Media Cybernetics, MD, United States).

In order to measure total mucins areas and quantities of goblet cells, tissue were stained with Alcian blue periodic acid Schiff staining kit (AB-PAS) (Solarbio, Beijing, China) after deparaffinization. The images were taken by a light microscope (Nikon Eclipse Ci-L, 200× magnification). The total mucins areas and quantities of goblet cells in epithelial cells of the jejunum tissues were counted by Image pro plus software 6.0.

### Immunohistochemistry and Immunofluorescence

Immunohistochemistry and immunofluorescence analysis were performed according to previous report from Ying et al. (26). For immunofluorescence, after deparaffinization, tissue sections were incubated with anti-mouse IgA (1:100) (Bioss, Beijing, China) primary antibody and conjugated FITC goat anti-rabbit antibody (1:400) for overnight. Then, DAPI (2 μg/ml) was used for nucleus staining. The images were taken by a fluorescence microscope (Nikon Eclipse Ci-L, 200× magnification). The total quantities of IgA-secreting cells were counted by Image pro plus software 6.0.

For immunohistochemistry, after deparaffinization, tissue sections were blocked with 5% BSA and stained with primary antibodies anti-mouse CD4 (1:200) (Servicebio, Wuhan, China) overnight at 4°C. Further, sections were treated with secondary antibody, peroxidase-conjugated goat anti-rabbit IgG secondary antibody (Bio-Rad, Hercules, CA, USA) with and then signals were detected using diaminobenzidine. The total quantities of CD4^+^T cells were counted by Image pro plus software 6.0.

### Western Blot Analysis

Western blot analysis was performed according to previous report from Ying et al. (26). In short, small intestinal tissues (50 mg) were homogenized on ice in RIPA lysis buffer (Servicebio, Wuhan, China). Homogenate was rotated, and the supernatant was collected. The total protein concentration was measured by a BCA protein assay kit. Equal amount of protein (20–40 µg) were loaded and separated by SDS-PAGE, transferred to polyvinylidene difluoride membranes (Millipore, MA), and blocked with 7.5% non-fat milk. Membranes were blotted with primary antibodies, anti-mouse β-actin (1:3000), anti-mouse claudin-1(1:1000), anti-mouse occludin (1:1000) or anti-mouse ZO-1 (1:1000) (Servicebio, Wuhan, China), overnight at 4°C, followed by 2 h incubation with horseradish peroxidase conjugated secondary antibody. Immunoreactive proteins were detected by ECL plus Western Blotting Detection System and quantified by densitometric analysis (Geldoc 2000 image, BioRad, USA).

### Gut Microbiota Analysis

DNA was extracted from fecal samples (50–100 mg) and analyzed according to previous reports of Liu et al. (27). DNA from different samples was extracted using the E.Z.N.A. ^®^Stool DNA Kit (D4015, Omega, Inc., USA) according to manufacturer’s instructions. The microbiota composition was assessed by PCR targeting the V3-V4 region of the bacterial 16S rRNA gene with the primer 338F-806R (fwd 5′-ACTCCTACGGGAGGCAGCAG-3′ and rev 5′-GGACTACHVGGGTWTCTAAT-3′). The PCR products were purified by AMPure XT beads (Beckman Coulter Genomics, Danvers, MA, USA) and quantified by Qubit (Invitrogen, USA). The amplicon pools were prepared for sequencing, and the size and quantity of the amplicon library were assessed on Agilent 2100 Bioanalyzer (Agilent, USA) and with the Library Quantification Kit for Illumina (Kapa Biosciences, Woburn, MA, USA), respectively. The libraries were sequenced on NovaSeq PE250 platform.

Samples were sequenced on an Illumina NovaSeq platform according to the manufacturer’s recommendations, provided by LC-Bio Technology Co., Ltd (Hangzhou, Zhejiang Province, China). Paired-end reads were assigned to samples based on their unique barcode and truncated through cutting off the barcode and primer sequence, then were incorporated using FLASH (28). According to fqtrim (V 0.94), clean tags were acquired by the quality filtering on the raw reads. Chimeric sequences were filtered using Vsearch software (v2.3.4). Feature table and feature sequence were obtained after dereplicated by DADA2. The complexity of species diversity of each sample was reveal by alpha diversity, while Beta diversity evaluated the differences of samples in the complexity of species. Alpha diversity and beta diversity were calculated by normalized to the same sequences randomly with QIIME2. Refer to SILVA (release 132) classifier, feature abundance was normalized using relative abundance of each sample. Blast was used for sequence alignment, and the feature sequences were annotated with SILVA database for each representative sequence.

### Fecal Metabolites Assessment

For fecal samples, refer to previous reports of Li et al. (29), 50 mg of lyophilized feces were extracted with 50% methanol buffer. The supernatants were further transferred into new 96‐well plates and were stored at −80°C prior to the LC‐MS analysis. All chromatographic separations were performed using an ultra-performance liquid chromatography (UPLC) system (SCIEX, UK). An ACQUITY UPLC T3 column (100 mm × 2.1 mm, 1.8 µm, Waters, UK) was used for the reversed phase separation. The column temperature was maintained at 35°C and the flow rate was set as previously described (30). A high-resolution tandem mass spectrometer TripleTOF5600plus (SCEIX, UK) was used to detect metabolites eluted from the column. The Q-TOF was operated in both positive (5 kV) and negative ion (−4.5 kV) modes. The mass spectrometry data were acquired in Interactive Disassembler Professional (IDA) mode, while the time-of-flight (TOF) mass ranged from 60 to 1200 Da (31). For the acquisition, the mass accuracy was calibrated every 20 samples, and a quality control (QC) samples was acquired from every 10 samples.

The acquired MS data was further processed by XCMS, CAMERA, and metaX toolbox with R software. Each ion was identified according to its retention time (RT) and m/z data. The metabolites were annotated through matching the precise molecular mass data (m/z) between sample and database (differences < 10 ppm) in Human Metabolome Database (HMDB) and online Kyoto Encyclopedia of Genes and Genomes (KEGG) database. The molecular formula of the metabolites would be identified and validated by the isotopic distribution measurements, while an in-house fragment spectrum library was used to validate the metabolite identification. MetaX further preprocess the intensity of peak data. Supervised OPLS-DA between two groups was conducted though metaX to distinguish the different variables.

### Statistical Analysis

The results of biological assay are presented as means ± SD. The differences between two groups were analyzed by Student’s *t* test. Multiple group comparisons were analyzed using one-way analysis of variance (ANOVA) with Bonferroni correction. All results were considered statistically significant at *P* < 0.05. The LEfSe method and the Kruskal-Wallis (KW) rank sum test was performed to identify features characterizing significant differences between two assigned classes. A value of LDA > 3.2 and *P* < 0.05 was considered statistically significant. Analysis of differential expression of metabolites was performed using *P* values and VIP values conducted by OPLS-DA. Correlations of genus in fecal flora and the fecal metabolites were determined by Spearman’s rank correlation tests.

## Results

### AG Restored Intestinal Immune Disorder

#### AG Treatments Adjusted Hematological Indices in CTX-Induced Immunosuppression Mice

**Figure 1:**
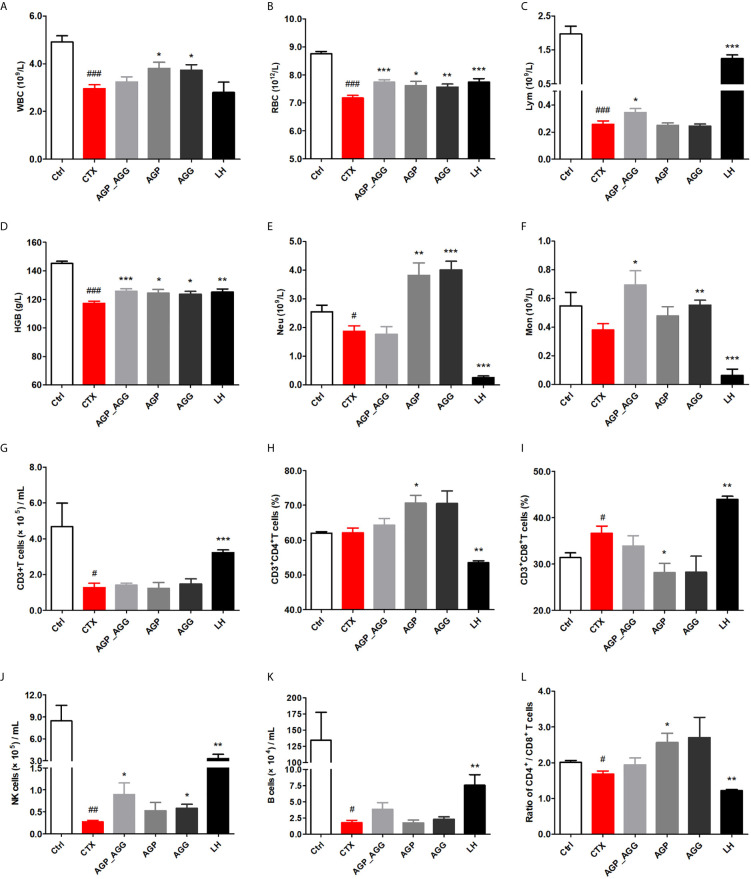
AG treatments adjusted hematological indices in CTX-induced immunosuppression mice. (n=7 for each group). **(A)** WBC. **(B)** RBC. **(C)** Lym. **(D)** HGB. **(E)** Neu. **(F)** Mon. **(G)** The quantity of CD3^+^T cells. **(H)** The percentage of CD3^+^CD4^+^T cells. **(I)** The percentage of CD3^+^CD8^+^T cells. **(J)** The quantity of NK cells. **(K)** The quantity of B cells. **(L)** The ratio of splenic CD4^+^/CD8^+^ T cells. Data are expressed as mean with SD. ^#^ *P* < 0.05, ^##^ *P* < 0.01, ^###^ *P* < 0.001 compared with Ctrl;*P < 0.05, **P < 0.01, ***P < 0.001 compared with CTX by student *t* test. Ctrl, control group; CTX, cyclophosphamide-induced immunosuppressive group; AGP_AGG, American ginseng polysaccharide+American ginseng ginsenoside with cyclophosphamide-induced immunosuppressive group; AGP, American ginseng polysaccharide with cyclophosphamide-induced immunosuppressive group; AGG, American ginseng ginsenoside with cyclophosphamide-induced immunosuppressive group.

In this study, hematological indices with significant differences among groups were performed (**Figures 1A–F**). White blood cells were consisted of lymphocytes (T cells and B cells), granulocytes (neutrophils, eosinophils, and basophils), and monocytes. Compared to control group, WBC, RBC, Lym, HGB (*P *<0.001) were significant reduced and Neu (*P *<0.05) decreased by CTX-treatment. All groups showed different degrees of repair by AG after CTX-induced immunotoxicity. AGP_AGG group has apparently increased Lym, Mon (*P *<0.05) and RBC, HGB (*P *<0.001). AGP group has significant increased WBC, RBC, HGB (*P *<0.05) and Neu (*P *<0.01). AGG group has significant increased WBC, HGB (*P *<0.05), RBC, Mon (*P *<0.01) and Neu (*P *<0.001).

Previous reports also shown the lymphocyte cell line is one of the most susceptible cell lines to the CTX (32). To further investigate the effect of AG on lymphocyte phenotypes, peripheral blood lymphocytes were investigated by determining the number of CD3^+^T cells, CD4^+^T cells, CD8^+^T cells, NK cells, and B cells by flow cytometry (**Figures 1G–L**). Compared to control group, the number of the B cells, CD3^+^T cells, and CD4^+^/CD8^+^ ratio (*P *<0.05) have been decreased by CTX-treatment, while the percentage of CD3^+^CD8^+^T cells (*P *<0.05) has been increased by CTX treatment, respectively. In particular, NK cells has been significantly decreased by CTX treatment compared to control group (*P *<0.01). Thereinto, compared to CTX group, AGP_AGG and AGG treatment have partially reverse the reduction of NK cells (*P *<0.05), while AGP treatment has significant increased the percentage of CD3^+^CD4^+^T cells and the CD4^+^/CD8^+^ ratio (*P *<0.05).

#### AG Treatments Altered Immune Organ Indices in CTX-Induced Immunosuppression Mice

**Figure 2:**
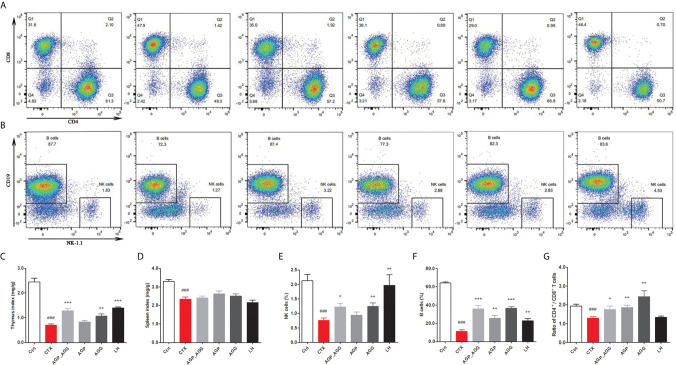
AG treatments altered immune organ indices in CTX-induced immunosuppression mice. (n=7 for each group). **(A)** The proportion of splenic CD4^+^ CD8^+^T cells was detected by flow cytometry. **(B)** The proportion of splenic NK1.1^+^ CD19^+^ cells was detected by flow cytometry. **(C)** Thymus index. **(D)** Spleen index. **(E)** The proportion of splenic NK cells. **(F)** The proportion of splenic B cells. **(G)** The ratio of splenic CD4^+^/CD8^+^ T cells. Data are expressed as mean with SD. ^###^ *P* < 0.001 compared with Ctrl; *P < 0.05, **P < 0.01, ***P < 0.001 compared with CTX by student *t* test. Ctrl, control group; CTX, cyclophosphamide-induced immunosuppressive group; AGP_AGG, American ginseng polysaccharide+American ginseng ginsenoside with cyclophosphamide-induced immunosuppressive group; AGP, American ginseng polysaccharide with cyclophosphamide-induced immunosuppressive group; AGG, American ginseng ginsenoside with cyclophosphamide-induced immunosuppressive group.

Additionally, compared to control group, CTX-treated mice significant decreased thymus and spleen indices (*P *<0.001), respectively (**Figures 2A, B**). There were no differences in spleen indices among other groups compared to CTX group (**Figure 2D**). In thymus indices (**Figure 2C**), compared to CTX group, AGG group rose (*P *<0.01), while AGP_AGG rose significantly (*P *<0.001).

The ratios of different splenic lymphocytes also have been investigated by flow cytometry. As shown in **Figures 2E–G**, splenic and peripheral blood lymphocytes have very similar tendency. The percentage of the NK cells, B cells, CD4^+^/CD8^+^ ratio have decreased by CTX-treatment (*P *<0.001) compared to control group, respectively. Furthermore, compared to CTX group, the percentages of NK cells was activated by AGP_AGG (*P *<0.05) and AGG treatment (*P *<0.01), the percentage of B cells was rose by AGP_AGG (*P *<0.001), AGP (*P *<0.01), and AGG treatment (*P*<0.001), while the CD4^+^/CD8^+^ ratio was increased by AGP_AGG (*P *<0.05), AGP (*P *<0.01), and AGG treatment (*P *<0.01).

#### AG Treatments Relived Intestinal Immune Disorder in CTX-Induced Immunosuppression Mice

**Figure 3:**
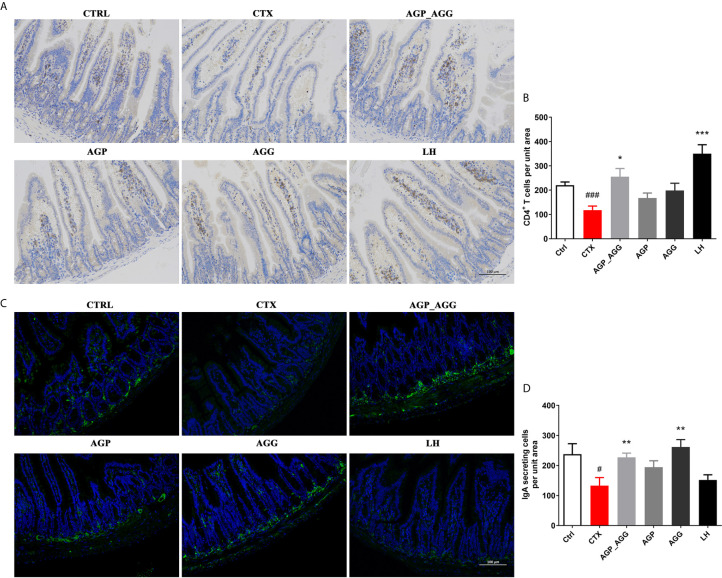
AG treatments relived intestinal immune disorder in CTX-induced immunosuppression mice. (n=5 for each group). **(A, B)** Immunohistochemical staining with CD4^+^ of jejunum section (200 ×, Scale bar = 100 μm). **(C, D)** Immunofluorescence staining with IgA^+^ and DAPI of jejunum section (200 ×, Scale bar = 100 μm). Data are expressed as mean with SD. ^#^ *P* < 0.05, ^###^ *P* < 0.001 compared with Ctrl; *P < 0.05, **P < 0.01, ***P < 0.001 compared with CTX by student *t* test. Ctrl, control group; CTX, cyclophosphamide-induced immunosuppressive group; AGP_AGG, American ginseng polysaccharide+American ginseng ginsenoside with cyclophosphamide-induced immunosuppressive group; AGP, American ginseng polysaccharide with cyclophosphamide-induced immunosuppressive group; AGG, American ginseng ginsenoside with cyclophosphamide-induced immunosuppressive group.

The gastrointestinal tract also regarded as an organ of immunity that defense the damage from viruses, bacteria, and parasites. Immunohistochemistry and immunofluorescence were used to determine the number of CD4^+^T cells (**Figures 3A, B**) and IgA-secreting cells (**Figures 3C, D**) in small intestinal tissue, respectively. In contrast to control group, the number of CD4^+^T cells (*P *<0.001), and IgA-secreting cells (*P *<0.05) were significant decreased by CTX treatment. After AGP_AGG treatment, this group has promoted the formation of CD4^+^T cells (*P *<0.05) and IgA-secreting cell (*P *<0.01) compared to CTX group. AGG group has recovered the number of IgA-secreting cell (*P *<0.01) after CTX treatment.

### AG Treatments Alleviated Gut Barrier Disruption in CTX-Induced Mice

**Figure 4:**
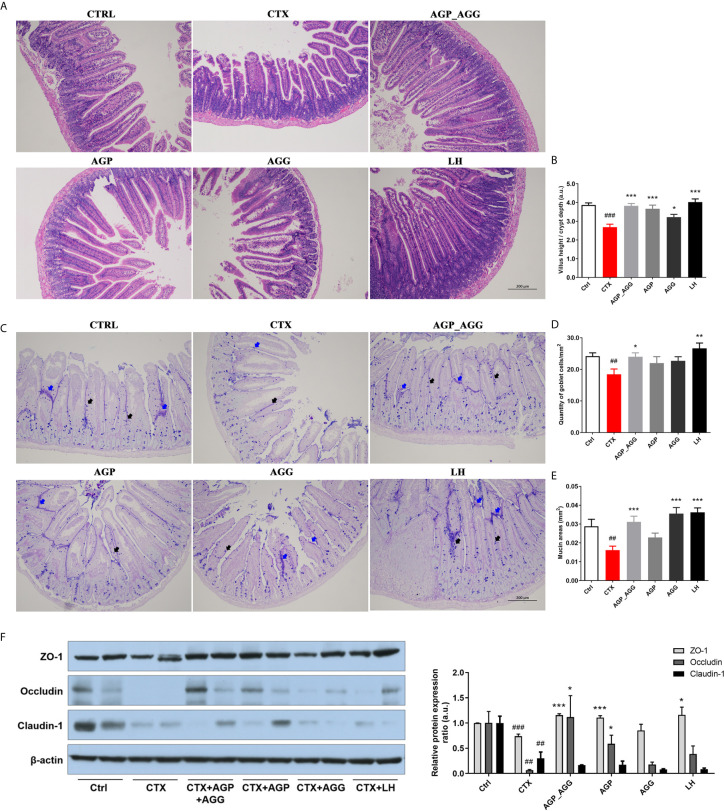
AG treatments alleviated gut barrier disruption in CTX-induced mice. (n=5 for each group). **(A)** HE staining of jejunum section (100 ×, Scale bar = 200 μm). **(B)** The ratio of villus height/crypt depth **(C)** AB-PAS staining of jejunum section. Black arrow represents goblet cells, while blue arrow represents mucins (100 ×, Scale bar = 200 μm). **(D)** The quantity of goblet cells/mm^2^ **(E)** Mucin areas (mm^2^). **(F)** Western blot assay of the protein level of ZO-1, occludin, and claudin-1 in jejunum section. Data are expressed as mean with SD. ##*P* < 0.01, ###*P* < 0.001 compared with Ctrl;*P < 0.05, **P < 0.01, ***P < 0.001 compared with CTX by student *t* test. Ctrl, control group; CTX, cyclophosphamide-induced immunosuppressive group; AGP_AGG, American ginseng polysaccharide+American ginseng ginsenoside with cyclophosphamide-induced immunosuppressive group; AGP, American ginseng polysaccharide with cyclophosphamide-induced immunosuppressive group; AGG, American ginseng ginsenoside with cyclophosphamide-induced immunosuppressive group.

Previous paper has reported that the gastrointestinal damage of chemotherapy or high dose CTX treatment can lead to apoptosis of intestinal crypts. It induces the reduction of the villus height and crypt length (33, 34). According to the histological section (**Figures 4A, B**) of jejunum, CTX induced serious intestinal mucosa damage, which displayed obvious cell infiltration and edema. Also, the gland pattern has been impaired with lower villus height (VH)/crypt depth (CD) ratio compared to control group. The CTX plus different AGP_AGG (*P *<0.001), AGP (*P *<0.001) and AGG treatment (*P *<0.05) significant increased VH/CD ratio, which showed moderate intestine injury compared to CTX group.

The AB-PAS stained jejunum sections were showed in **Figures 4C–E**, which have the similar tendency toward HE-stained jejunum sections. The intestine structural integrity was destroyed by CTX with fewer goblet cells *P *<0.01 and mucin areas (*P *<0.01) compared to control group. However, AGP_AGG treatment partially reversed the number of goblet cells (*P* <0.05) and mucin areas (*P *<0.001) compared to CTX group.

Tight junction associated proteins including integral transmembrane proteins (e.fg. occludin, claudins), peripheral membrane adaptor proteins (e.g. zonula occludens) are explored in this study (**Figure 4F**). ZO-1 (*P *<0.001), occludin (*P *<0.01), and claudin-1 (*P *<0.01), these proteins have been significant decreased by CTX-treatment compared to control group. For ZO-1 and occludin proteins, both of the AGP_AGG and AGP group have similar effects in increasing the protein contents (*P *<0.001 for ZO-1, *P *<0.05 for occludin), which alleviated CTX-induced damage in tight junction proteins.

#### AG Attenuated Gut Dysbiosis in CTX-Induced Mice

**Figure 5:**
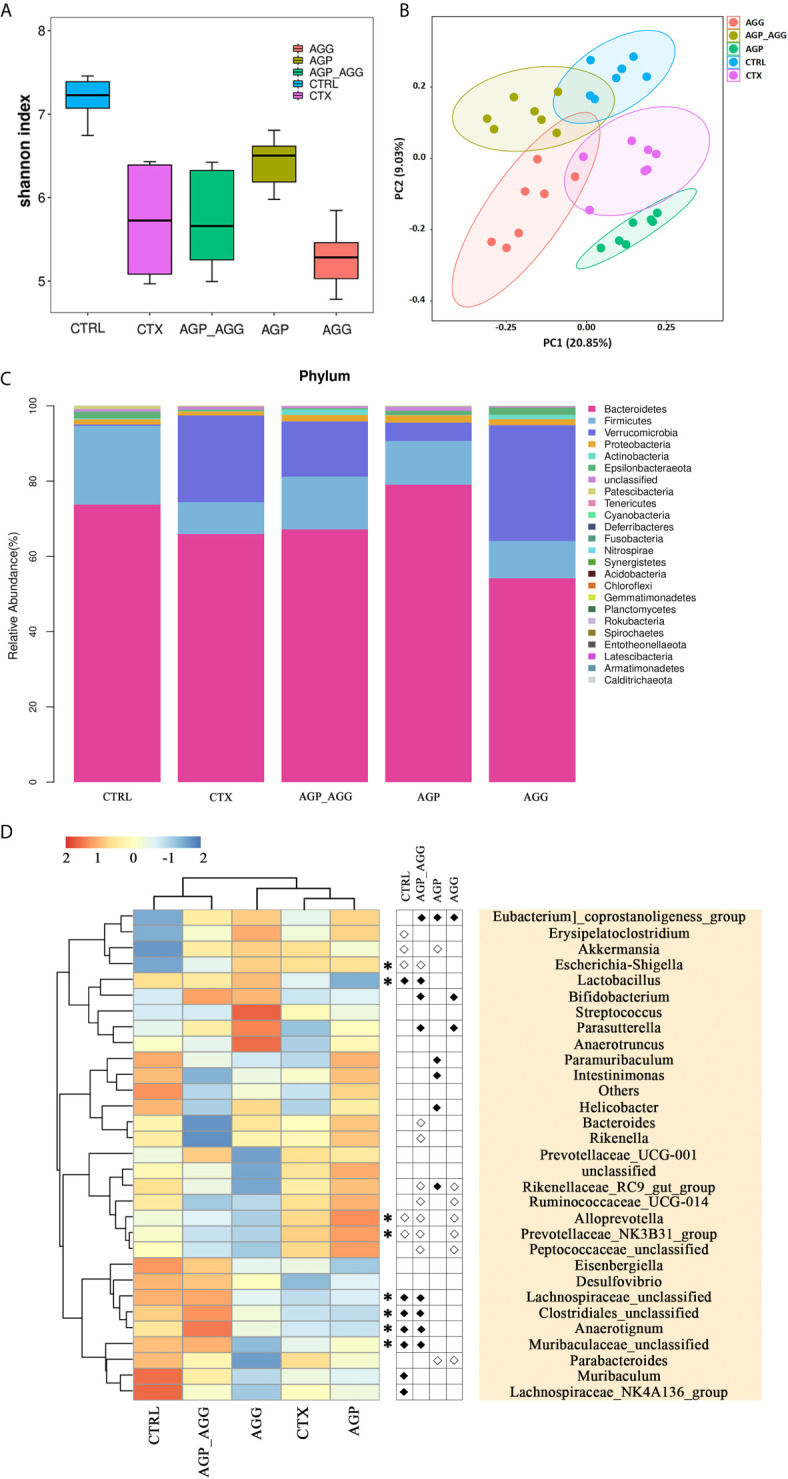
AG treatments attenuated gut dysbiosis in CTX-induced mice. (n=7 for each group). **(A)** Alpha-diversity estimators by Shannon indexes. Data are expressed as mean with SD. **(B)** Unweighted UniFrac distance-based principal coordinate analysis. **(C)** Bacterial taxonomic profiling at the phylum level. **(D)** Heat map of relative abundance of top 30 genus. In the middle panel, the differences of abundance distributions among genera between two groups were measured by the Wilcoxon rank-sum test, *P <*0.05. White circles represent less abundant genera in CTRL, AGP_AGG, AGP, and AGG compared with CTX; Black diamonds represent more abundant genera in CTRL, AGP_AGG, AGP, and AGG compared with CTX. Asterisks represent genera whose abundance in CTRL mice was altered by CTX and then regulated by AGP_AGG. CTRL, control group; CTX, cyclophosphamide-induced immunosuppressive group; AGP_AGG, American ginseng polysaccharide+American ginseng ginsenoside with cyclophosphamide-induced immunosuppressive group; AGP, American ginseng polysaccharide with cyclophosphamide-induced immunosuppressive group; AGG, American ginseng ginsenoside with cyclophosphamide-induced immunosuppressive group.

The high-throughput 16S rDNA pyrosequencing of fecal samples was analyzed to illustrate the regulatory effect of the AG on the gut microbiota. The common microbial α-diversity indices, such as Shannon index, has shown in **Figure 5A**. The Shannon index was decreased by CTX-treatment (*P *<0.001) in relative to control group. After AGP treatment, microbial community diversity and uniformity mildly reversed (*P <*0.05) compared with CTX group. To visualize the overall microbial community structure differences, the β-diversity of microbial composition was calculated using unweighted UniFrac-based PCoA (**Figure 5B**). It was obviously separated among CTX group and other groups, especially AGP group.

The taxonomic composition of each group in phylum and genus level was analyzed. At the phylum level, *Bacteroidetes, Firmicutes, Verrucomicrobia* make up more than 90 percent of the microbiota among all groups (**Figure 5C**). In this study, CTX group decreased the abundance of *Bacteroidetes* (*P >*0.05) and *Firmicutes* (*P <*0.05) compared to control group. In contrast to CTX group, the abundance of *Bacteroidetes* significantly increased in AGP group (*P *<0.001). *Bacteroidetes has* been regarded as primary degraders of varieties of fiber polysaccharide, while *Firmicutes* tends to be particular for a select set of glycan (35). In genus level, the heatmap showed the top 30 genera among all groups (**Figure 5D**). AGP and AGG group has different effects on relative abundances of microbial community composition, while AGP-AGG co-treated group has taken the advantage of both groups that has a closer evolutionary relationship to control group. By student *t* test, compared to CTX group, the genera of *Eubacterium]_coprostanoligenes_group, Lactobacillus, Bifidobacterium, Parasutterella, Lachnospiraceae_unclassified, Clostridiales_unclassified, Anaerotignum, Muribaculaceae_unclassified* were increased and were accompanied by decreased in the genera of *Escherichia−Shigella, Bacteroides, Rikenella, Rikenellaceae_RC9_gut_group, Ruminococcaceae_UCG−014, Alloprevotella, Prevotellaceae_NK3B31_group*, and *Peptococcaceae_unclassified*, after AGP-AGG treatment (*P <*0.05). Among them, AGP_AGG intervention significant reversed the relative abundance of eight genera (five up-regulated and three down-regulated, Asterisks in **Figure 5D**) that induced by CTX-treatment.

**Figure 6:**
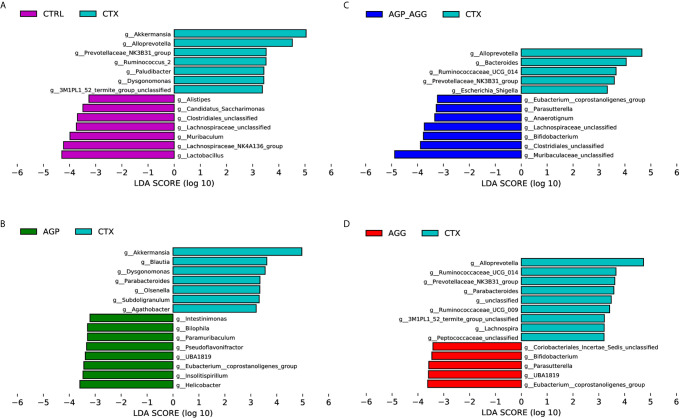
Bacterial communities’ differences obtained by linear discriminant analysis (LDA) analysis. **(A)** LDA results between the CTRL and CTX groups. **(B)** LDA results between the AGP_AGG and CTX groups. **(C)** LDA results between the AGP and CTX groups. **(D)** LDA results between the AGG and CTX groups. Only the genus level with a significant logarithmic LDA threshold score of > 3.2, Wilcoxon rank-sum test, p < 0.05 were shown. Different-colored regions represent different groups. CTRL, control group; CTX, cyclophosphamide-induced immunosuppressive group; AGP_AGG, American ginseng polysaccharide+American ginseng ginsenoside with cyclophosphamide-induced immunosuppressive group; AGP, American ginseng polysaccharide with cyclophosphamide-induced immunosuppressive group; AGG, American ginseng ginsenoside with cyclophosphamide-induced immunosuppressive group.

To find the key phylotypes and biomarkers of gut microbiota among various groups, the LDA analysis is displayed (**Figure 6**). Among the cross-comparisons of different groups (CTRL vs CTX, AGP_AGG vs CTX, AGP vs CTX, AGG vs CTX), LDA results showed 55 discriminative features in genus level (LDA score >3.2, *P<*0.05). Compared to control group, CTX group increased *g_Akkermansia, g_Alloprevotella, g_Prevotellaceae_NK3B31_group, g_Ruminococcus_2, g_Paludibacter, g_Dysgonomonas, g_3M1PL1_52_termite_group_unclassified*, and decreased *g_Alistipes, g_Candidatus_Saccharimonas, g_Clostridiales_unclassified*, *g_Lachnospiraceae_unclassified, g_Muribaculum, g_Lachnospraceae_NK4A136_group, g_Lactobacillus.* In contrast to CTX group, AGP_AGG treatment enriched *g_Eubacterium_coprostanoligenes group, g_Parasutterella, g_Anaerotignum, g_Lachnospiraceae_unclassified, g_Bifidobacterium, g_Clostridiales_unclassified, g_Muribaculaceae_unclassified*, while decreased *g_Alloprevotella, g_Bacteroides, g_Ruminococcaceae_UCG-014, g_Prevotellaceae_NK3B31_group, g_Escherichia-Shigella.*

#### AG Treatments Alters Fecal Metabolites in CTX-Induced Mice

**Figure 7:**
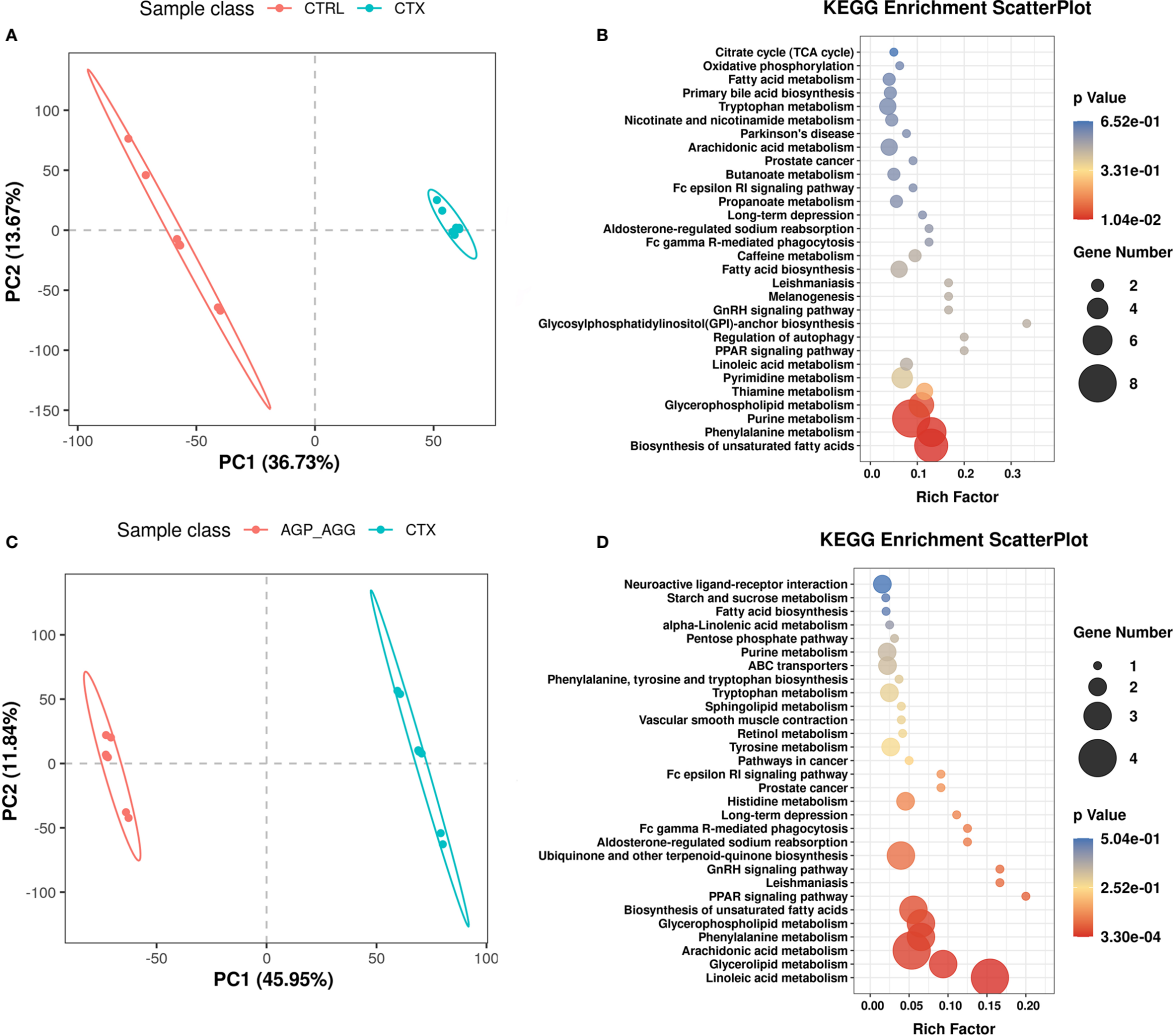
AG treatments alters fecal metabolites in CTX-induced mice. (n=7 for each group). **(A)** OPLS-DA score plots from CTRL group vs CTX group. **(B)** Pathway Enrichment based on altered metabolites from CTRL group vs CTX group. **(C)** OPLS-DA score plots from AGP_AGG group vs CTX group. **(D)** Pathway Enrichment based on altered metabolites between AGP_AGG group and CTX group. CTRL, control group; CTX, cyclophosphamide-induced immunosuppressive group; AGP_AGG, American ginseng polysaccharide+American ginseng ginsenoside with cyclophosphamide-induced immunosuppressive group.

The complex interactions between host and gut microbiota are intense associated with host-microbe metabolic axes. Notably, gut microbiota plays an essential role in immunometabolism through their metabolites such as short-chain fatty acids, bile acids, and amino acids (36). Hence, the untargeted metabolomics analysis was generated on fecal samples by ultra-high performance liquid chromatography-quadrupole time-of flight mass spectrometry (UPLC-QTOF/MS). There were overall 680 and 979 metabolites has been identified in feces, under negative and positive mode, respectively. The orthogonal partial least squares–discrimination analysis (OPLS-DA) was applied to distinct control group with CTX group (CTRL vs CTX, **Figure 7A**), as well as the AG supplement group with CTX group (AGP_AGG vs CTX, **Figure 7C**; AGP vs CTX, **Figure S1A**; AGG vs CTX, **Figure S1D**). The R^2^ and Q^2^ of OPLS-DA score analysis were summarized in **Table S2**, validating the good classification for the model. Metabolites with *VIP* values >1.0 and *P* value <0.05 were considered significantly change. These altered metabolites were further generated the KEGG metabolic pathways by MetaboAnalyst (www.metaboanalyst.ca) between CTRL vs CTX (**Figure 7B**), AGP_AGG vs CTX (**Figure 7D**), AGP vs CTX (**Figure S1B**), AGG vs CTX (**Figure S1D**), respectively. Compare between control group and CTX group, biosynthesis of unsaturated fatty acids, phenylalanine metabolism, purine metabolism, glycerophospholipid metabolism, thiamine metabolism, and pyrimidine metabolism (*P <*0.05) were significant enriched. Linoleic acid metabolism, glycerolipid metabolism, phenylalanin metabolism, arachidonic acid metabolism, and biosynthesis of unsaturated fatty acids (*P <*0.05) were the markedly enriched between AGP_AGG and CTX group. By cross-comparisons of AGP_AGG vs CTX and CTX vs CTRL group, both CTX group and AGP_AGG group has great effects on biosynthesis of unsaturated fatty acids, phenylalanin, purine, and glycerophospholipid metabolism. It implicated AGP_AGG group might partially reverse some of the side effects of CTX-treated through these metabolisms.

**Figure 8:**
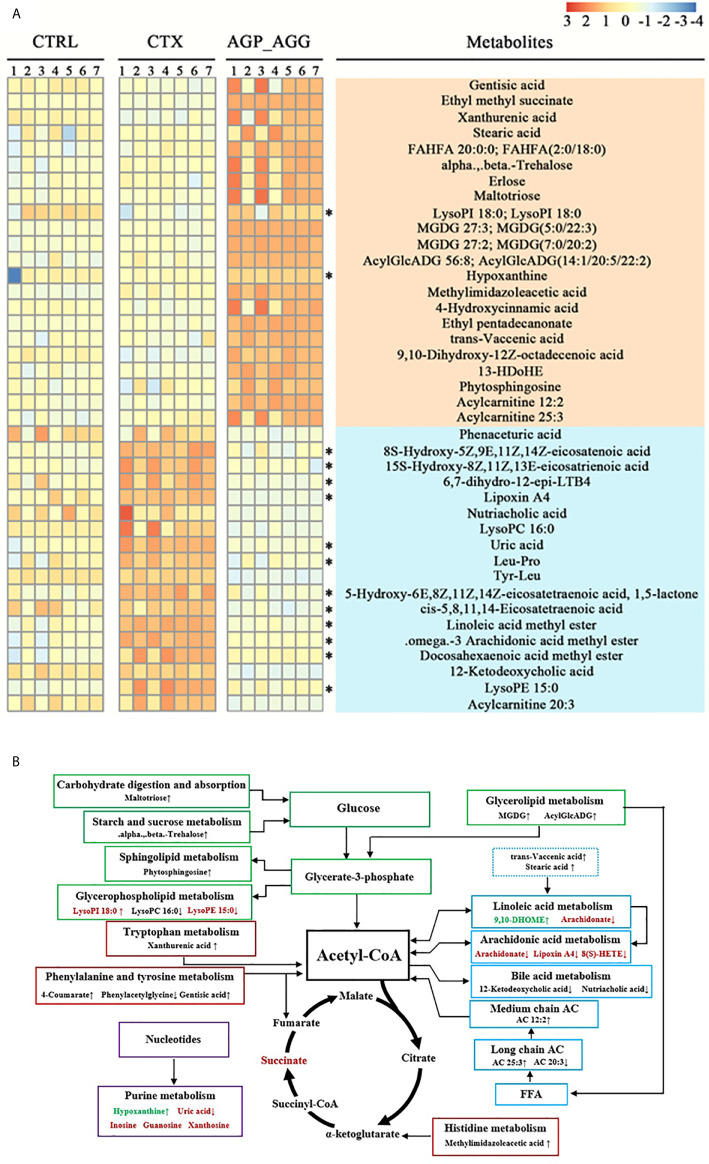
Heatmap and metabolic network analysis of the identified metabolites. **(A)** Heatmaps of the differential metabolites that were altered by CTX compared with AGP_AGG mice. Asterisks represent metabolites whose abundance in CTRL mice was altered by CTX and then regulated by AGP_AGG. **(B)** The profile of metabolic network analysis of the identified metabolites based on the known metabolic pathways. Red color represents significantly increased metabolites in CTX group, while green represents decreased dramatically, compared to CTRL group. “↑↓” describes metabolites that were significantly up- or down-regulated after AGP_AGG treatment. FFA, free fatty acid; AC, acylcarnitine; CTRL, control group; CTX, cyclophosphamide-induced immunosuppressive group; AGP_AGG, American ginseng polysaccharide+American ginseng ginsenoside with cyclophosphamide-induced immunosuppressive group.

To further illustrate the biomarkers of AGP_AGG treatment group, the endogenous metabolites with fold change > 3 or <1/3 was selected as important features. The cross-comparisons of different groups were listed (**Table S3**). CTX treatment has triggered extensive change in endogenous metabolites, in which 46 increased and 9 decreased, respectively, compared with control group. CTX treatment significant increased the metabolites related to purine metabolism (guanosine, inosine, uric acid, xanthosine), propanoate metabolism (succinic acid, propionic acid), and decreased the metabolites associated with thiamine metabolism (thiamine, 5-(2-Hydroxyethyl)-4-methylthiazole). AGP_AGG treatment has induced 22 metabolites and 18 repressed metabolites compared with CTX group (**Figure 8A**). 24 (60.0%) of differential metabolites between AGP_AGG treatment and CTX group were lipid and lipid-like molecules, which involved in arachidonic acid metabolism, linoleic acid metabolism, glycerophospholipid metabolism, and so on. AGP_AGG treatment has partially abrogated 14 metabolites change (12 down-regulated and 2 up-regulated) induced upon CTX-treatment (asterisks in **Figure 8A**). Taken together, the remarkable change of metabolites was summarized in **Figure 8B**, which involved in amino acids, fatty acids, and carbohydrate metabolisms.

**Figure 9:**
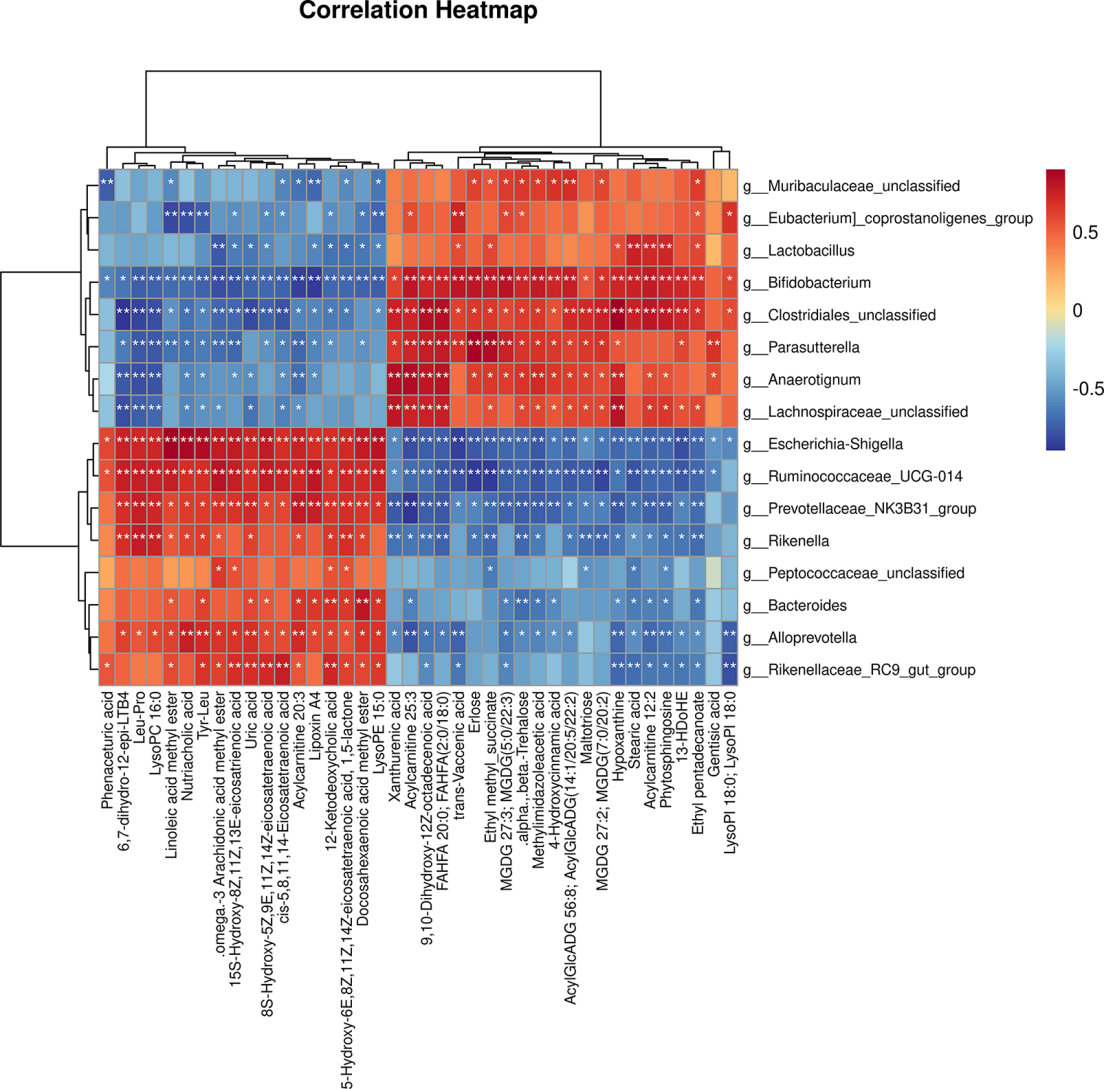
Correlation analysis of the gut microbiome and fecal metabolites as well as relationship. Spearman’s rank correlation between 16 most differential genera and 40 differential metabolites from AGP_AGG group vs CTX group. *P<0.05, **P<0.01 denoted statistical significance between bacterial taxa and metabolites.

As shown in **Figure 9**, the 16 most differential fecal microbiota in genus level and 40 differential metabolites were conducted the Spearman’s correlation analysis to identify microbes-metabolites relationship upon AGP_AGG treatment. Strong correlations (*P<*0.05) were observed in 436 microbes-metabolites pairs. Metabolites such as uric acid, was significant decreased after AGP_AGG treatment and showed a strong positive correlation (*P<*0.01) with *g_Alloprevotella, g_Escherichia-Shigella, g:Prevotellaceae_NK3B31_group, g:Rikenellaceae_RC9_gut_group, g:Ruminococcaceae_UCG-014*, and a strong negative correlation (*P<*0.01) with *g_Bifidobacterium, g_Clostridiales_unclassified*. The increased metabolites such as 9,10-Dihydroxy-12Z-octadecenoic acid, acylcarnitine 25:3, and FAHFA 20:0; FAHFA(2:0/18:0) that related to energy metabolism were positively correlated with *g_Bifidobacterium, g_Clostridiales_unclassified, g_Anaerotignum, g_Lachnospiraceae_unclassified, g_Parasutterella* (*P<*0.01), and negatively correlated with *g_Escherichia-Shigella, g_Ruminococcaceae_UCG-014, g_Prevotellaceae_NK3B31_group, g_Rikenella* (*P<*0.01).

## Discussion

CTX is an alkylating agent and widely used in cancer treatments. However, it has various toxicities not only in cancer cells but also in rapidly dividing cells, such as immune cells and epithelial cells. High dose of CTX treatment can lead to immunosuppressive effects, damage of intestinal epithelium, and dysfunction of gut microbiota (37, 38). AG is a general tonics to maintain human body homeostasis and also has been applied as immune-modulator in various diseases (2). Furthermore, both ginsenoside and polysaccharides of AG have been reported with their immunostimulating actions *in vitro* and vivo, respectively (12, 39, 40). In this study, we confirmed the immunomodulatory capacity of AG in mucosal, systemic immunity, and gut barrier protection effect using a CTX-induced immunosuppressed mice model, which co-treated with polysaccharide and ginsenoside.

Intestine is not only the largest digestion and absorption organ, but also involves mucosal immunity in human body that nourish up to 70% of the body’s lymphocytes population (41). CD4^+^T cells are essential for intestinal immune homeostasis by discriminating between harmless stimuli and harmful pathogens. CD4^+^T cells are mostly located in the lamina propria of intestine and participate in the immune response through the release of various pro- and anti-inflammatory cytokines and the secretion of co-stimulatory molecules. Furthermore, activated CD4^+^T cells can differentiate into various T helper cells with different effector functions, such as response in cancer immunotherapy (42, 43). IgA from B cells is the most prevalent immunoglobulin in the human body and is the essential effector molecules to protect mucosal surfaces against infections in the intestine. Hence, IgA synthesis and secretion has been regarded as the most recognized characteristic in mucosal immunity (44, 45). In this study, co-treated of AGP_AGG could alleviate mucosal immunosuppression by stimulating the formation of both CD4^+^T cells and IgA-secreting cells in small intestine.

The gastrointestinal tract mucosa is the largest interface that interacts between host and external environment. Besides being a physical barrier, the intestinal epithelial barrier communicates with gut microbiota and immune cells to maintain the gut homeostasis. In specific, goblet cells are particular epithelial cells in mucosal surface and maintain the mucosal barriers through the secretion of mucus. Also, goblet cells involve in mucosal immunity over secretion of anti-microbial proteins, chemokines, and cytokines (46). Tight junctions play essential roles in physical intestinal barrier and their dysfunction is highly associated with metabolic and inflammatory diseases (47). In this study, AG, especially AGP_AGG groups could restore the intestinal barrier damage in epithelial cells as well as tight junction proteins.

Our study showed the combination use of AG polysaccharide and ginsenoside has a better effect in modulation of mucosal immunity and protection of gut barrier. On the one hand, studies reported that ginsenosides Rb1 could stimulate the phagocytic capacity of macrophages for bacteria *via* activation of the Fc-gamma receptors and p38/Akt pathway (48), while the combination use of ginsenoside Rg3, Rk1, Rg5 could stimulate immune reactions through nuclear factor-kappa B (NF-κB) and cytokine secretion (49, 50). In addition, polysaccharides could potentially activate receptors like Toll-like receptors (TLRs), C-type lectin receptors, and so on (51), which are the key receptors related to intestinal immunity against pathogen (52, 53). On the other hand, many studies have suggested that even though polysaccharides is non-digestible, it was still used as immunomodulator. Non starch polysaccharides might be recognized as potential prebiotics altering the composition of gut microbiota or can be transformed into short chain fatty acids that further participate into immune pathways (54, 55). Commensal microorganisms are essential for the development and maturation of immune system. Also, prebiotics can reduce the plasma endotoxemia and improve the gut barrier integrity (56). Previous report demonstrated polysaccharide-mediated gut microbiota improved the metabolism and absorption of ginsenoside that lead to the synergistic effect of ginseng decoction (57).

To identify the crucial bacteria and the underlying mechanisms of the AG immuno-modulator actions, the gut microbiota in different levels were investigated. Consistent with previous reports, the AG polysaccharide restored the microbial community diversity and uniformity, together with the microbial community composition in phylum level. In genus level, AGP-AGG co-treated group has taken the advantage of polysaccharide and ginsenoside that similar to control group. Also, an increase of immunomodulatory potential bacteria and a decrease of pathogenic bacteria were observed in AGG_AGP group. LDA analysis also has been performed to identify the specific bacteria related to AGP_AGG, AGP, AGG, as well as CTX group. *Lactobacillus* and *Bifidobacterium* are well known probiotics for promoting mucosal immunity in enhancing CD^+^ T cell–dendritic cell interactions, lymphocyte proliferation, and cytokine secretion (58, 59). Also, these probiotics strengthen the barrier integrity by activating enterocytes express epithelial growth factor receptor (EGF-R), increasing intestinal mucins, and stimulating IgA production (60). *Parasutterella* has been regarded as a core component of the human and mouse gut microbiota and always positively correlated with health index (61). *Lachnospiraceae* is known as butyrate-producing bacteria and antagonize *Clostridium difficile* infection. Butyrate has been reported for its trophic effect on mucosa (62). *Eubacterium_coprostanoligenes*, a gram-positive coccobacillus, has been reported that contributed to cholesterol reduction (63). Selected strains of *Clostridiales* could induce Treg cells development independent of MyD88-signaling and might attribute to short-chain fatty acids (SCFAs) (64, 65). According to previous studies, cyclophosphamide can lead to disruption of the gut microbiota. Especially in high dose of cyclophosphamide leaded to increase various of pathogenic counts, such as *Escherichia coli* and so on (38). In this study, *Dysgonomonas* has been identified as the biomarker of CTX group in this study, which can cause gastroenteritis in immunocompromised patient (66). *Escherichia−Shigella* has been decreased after AGP_AGG treatment, which produces shigatoxin during infection and causes various life-threatening disease (67). *Peptococcaceae* may be or not being pathogenic and might relate to stress and diet (68).

The disordered metabolic environment induced by CTX treatment can lead to various side effects on gut. AGP_AGG treatment group corrected some disordered metabolites such as uric acid, lysoPE 15:0 and lysoPI 18:0. Purines act various functions in cells such as components of DNA and RNA, as energy sources. However, the abnormal elevated of the final product of purine metabolism in urine or serum, uric acid, is always related to gout, kidney, and vascular disorders (69) as well as damage of intestinal barriers (70). Lysophospholipids (LPLs) can act through G protein-coupled receptors and play essential roles in immune cell trafficking. However, dysfunction of LPLs, such as hyperlipidemia, can induce endothelial cell activation and injury (71). In addition, AGG_AGP varied many metabolites that have been implicated to have strong associations with immune cells or protective effect of gut dysbiosis. Immune cells utilize the same pathways as other cells to generate energy and make sure their proper functioning. Hence, immunometabolism, as a new branch of metabolism, notably amino acids, fatty acids, glucose, is essential needed for immune cells on homeostasis or pathological state. The metabolites of some amino acids, such as tryptophan, are related to cell proliferation and growth processes (71). Xanthurenic acid is one of tryptophan metabolites in the kynurenine pathway and regarded as aryl hydrocarbon receptors (AhR) ligands. AhR is an essential regulator of intestinal immunity, inflammation as well as maintenance of gut homeostasis (72). Fatty acid oxidation can be utilized as an energy production in mitochondria that also involved in immune cells. Aerobic glycolysis is a sign of activated CD8^+^T cells, both naive and memory T cells depend on oxidative phosphorylation. T native cells oxidize external lipids in mitochondrion and utilize as energy source, which found abundant acylcarnitine molecules in metabolomic analysis of these cells (73). 9, 10-DHOME is a derivative of linoleic acid diol as well as a proliferator-activated receptor (PPAR) gamma 2 ligand. PPAR-gamma is a lipid -activated transcription factor that regulates a host of gene related to lipid metabolism (73) and also regulate differentiation and development of immune cells, such as monocytes, T cells, and NK cells (74). The metabolites variations resulted by AGP_AGG supplement might also play a protective role in chemotherapy induced gut dysfunction. Numerous evidence has reported omega-3 PUFAs including docosahexaenoic acid (DHA) ameliorated inflammation and improved wound healing that related to chemopreventive potential in cancer (75). 13-HDoHE as an oxidation product of DHA also attracted much attentions for its possibility in anti-inflammation effect (76). The PC and lysophosphatidylcholines (LPC), can mutually transform to each other and have reported the increase in blood might be related to evoke of oxidative stress (77).

## Conclusion

**Figure 10:**
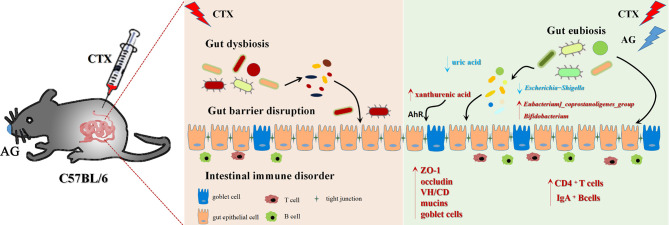
Summarized mechanisms underlying the immunomodulatory effect of AG on CTX induced intestinal immune disorder and gut barrier dysfunction.

In summary (**Figure 10**), our work investigated the immunostimulatory effects of the main constituents of AG. It suggests that AG effectively restored intestinal immune disorder, recovered impaired mucosal integrity, and attenuated intestinal microbiota dysfunction. In particular, this work revealed co-treated of AG polysaccharide and ginsenoside alleviated the side effects of CTX *via* regulating gut microbiota and related metabolism. The increase of benefit bacteria *Lactobacillus, Bifidobacterium, Parasutterella, Lachnospiraceae, Eubacterium_coprostanoligenes, Clostridiales* as well as metabolites xanthurenic acid, acylcarnitine, 9, 10-DHOME, 13-HDoHE were related to the therapeutic effects of AGP_AGG.

## Data Availability Statement

The 16S rRNA sequencing data has been uploaded to NCBI - Bioproject, ID: PRJNA702634.

## Ethics Statement

The animal study was reviewd and approved by the Institutional Animal Care and Use Committee (IACUC) of the Institute of Chinese Medicine, Hunan Academy of Chinese Medicine (Hunan, China).

## Author Contributions

RZ, HZ, HL, and LH conceived and designed the study. RZ, HZ, DH, and QZ performed the experiments. RZ, HZ, and JX analyzed the data, drawn the figure and written the manuscript. LH and HL revised the manuscript, obtained the funding, and supervised the whole study. All authors contributed to the article and approved the submitted version.

## Funding

This study was supported by Natural Science Foundation of China (81891013, 81891010) and Construction Project of Sustainable Use of Precious Chinese Medicine Resources (2060302).

## Conflict of Interest

The authors declare that the research was conducted in the absence of any commercial or financial relationships that could be construed as a potential conflict of interest.

## Supplementary Material

Supplementary Figure 1 AG treatments alters fecal metabolites in CTX-induced mice. (n=7 for each group). **(A)** OPLS-DA score plots from AGP group vs CTX group. **(B)** Pathway Enrichment based on altered metabolites from AGP group vs CTX group. **(C)** OPLS-DA score plots from AGG group vs CTX group. **(D)** Pathway Enrichment based on altered metabolites between AGG group and CTX group. CTX, cyclophosphamide-induced immunosuppressive group; AGP_AGG, American ginseng polysaccharide+American ginseng ginsenoside with cyclophosphamide-induced immunosuppressive group; AGP, American ginseng polysaccharide with cyclophosphamide-induced immunosuppressive group; AGG, American ginseng ginsenoside with cyclophosphamide-induced immunosuppressive group.
