# Supplementary figures and images for: The Synergistic Effects of Polysaccharides and Ginsenosides From American Ginseng (Panax quinquefolius L.) Ameliorating Cyclophosphamide-Induced Intestinal Immune Disorders and Gut Barrier Dysfunctions Based on Microbiome-Metabolomics Analysis
Source: Front Immunol. 2021 Apr 22;12:665901. doi: 10.3389/fimmu.2021.665901 (PMC8100215; doi:10.3389/fimmu.2021.665901)

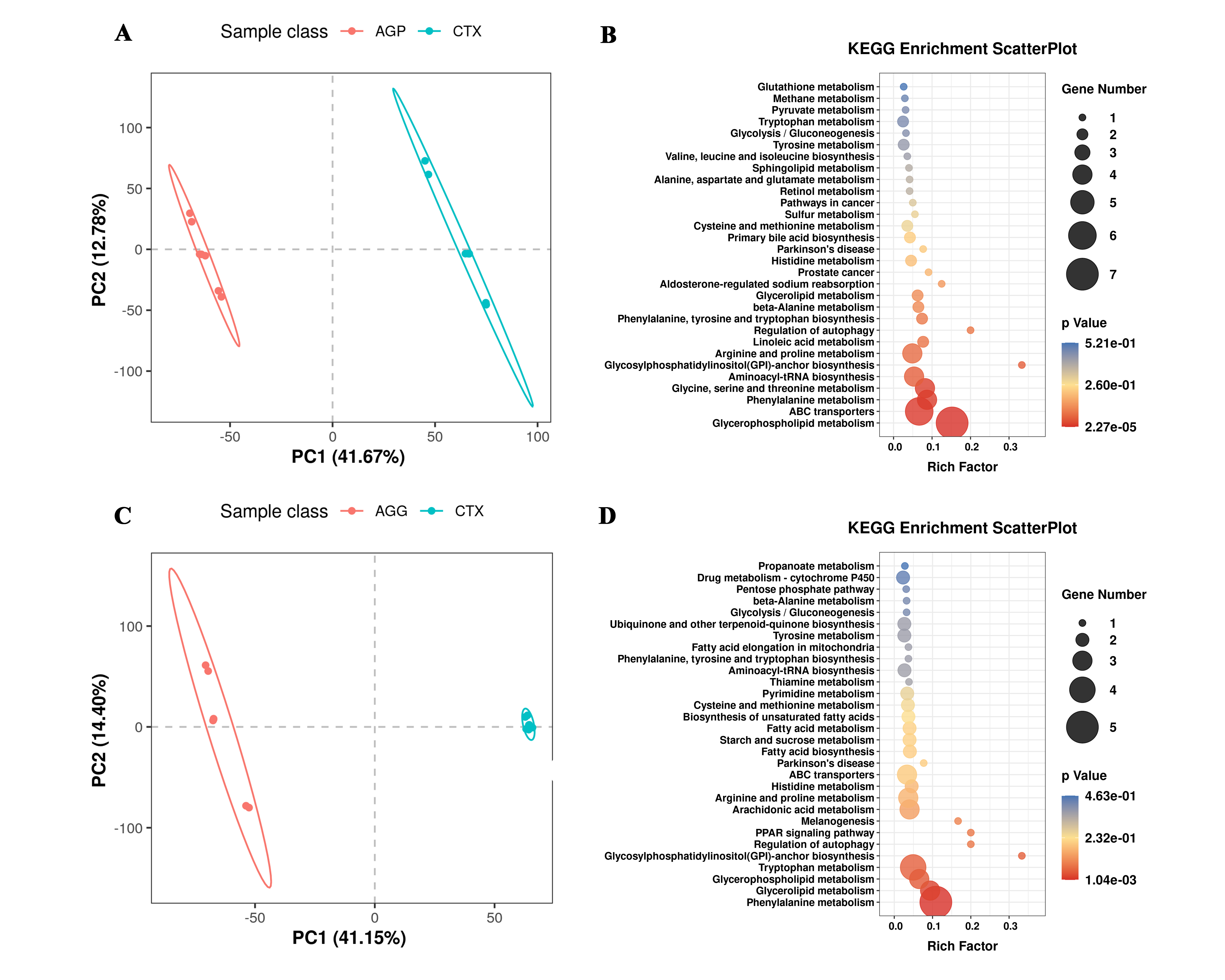

Supplement: Supplementary Figure 1 — AG treatments alters fecal metabolites in CTX-induced mice. (n=7 for each group). (A) OPLS-DA score plots from AGP group vs CTX group. (B) Pathway Enrichment based on altered metabolites from AGP group vs CTX group. (C) OPLS-DA score plots from AGG group vs CTX group. (D) Pathway Enrichment based on altered metabolites between AGG group and CTX group. CTX, cyclophosphamide-induced immunosuppressive group; AGP_AGG, American ginseng polysaccharide+American ginseng ginsenoside with cyclophosphamide-induced immunosuppressive group; AGP, American ginseng polysaccharide with cyclophosphamide-induced immunosuppressive group; AGG, American ginseng ginsenoside with cyclophosphamide-induced immunosuppressive group. [file Image_1.jpeg]
